# O Primeiro Estágio da Operação de Norwood no Brasil – Elevamos o Patamar

**DOI:** 10.36660/abc.20220420

**Published:** 2022-07-29

**Authors:** Walter Villela de Andrade Vicente

**Affiliations:** 1 Universidade de São Paulo Faculdade de Medicina de Ribeirão Preto Ribeirão Preto SP Brasil Universidade de São Paulo – Faculdade de Medicina de Ribeirão Preto, Ribeirão Preto, SP – Brasil

**Keywords:** Cardiopatias Congênitas, Síndrome do Coração Esquerdo Hipoplásico/cirurgia, Oxigenação por Membrana Extracorpórea – ECMO, Procedimento de Norwood/cirurgia

Os resultados do primeiro estágio da operação de Norwood no Brasil são pouco relatados, provavelmente, por contrastarem demasiadamente com os do mundo desenvolvido.

Contrariamente a esse cenário, nessa edição dos Arquivos Brasileiros de Cardiologia, o grupo de da Silva apresenta sua experiência em um único centro, com a maior série da operação de Norwood primeiro estágio, na qual foi utilizado suporte adjuvante pós-operatório com oxigenação extracorpórea (ECMO), em nosso país. A sobrevida relatada, de 91,3%, em 30 dias, um excelente resultado cirúrgico, é similar à de algumas instituições internacionais de elite nas quais a ECMO também está disponível.^1^

O Rubicão foi cruzado! Parabéns aos autores!

Considerando que a síndrome do coração esquerdo hipoplásico (SCEH) é a mais prevalente e uma das mais desafiadoras formas de ventrículo único, o referido relato repercutirá na comunidade brasileira de cardiologia pediátrica e em seus centros cirúrgicos de referência, no Sistema Único de Saúde (SUS), no setor privado de saúde e no público em geral.

Gostei muito do estudo. Ele inclui uma avaliação retrospectiva de 80 pacientes (privados, n=79; públicos, n=1) operados de 2016 a 2019. No período, o suporte com ECMO foi disponibilizado no hospital, um dos maiores centros de referência em cirurgia cardiovascular do país. O relato é minucioso e os dados, devidamente analisados.

É digno de nota que os autores reconhecem o fato de que os resultados se beneficiaram da experiência acumulada pelo grupo, refinada ao longo do tempo, e que abrange mais de 500 casos. Em outras palavras, estamos olhando para a ponta do iceberg de pacientes acumulados por da Silva.^1^

Foi adotado um protocolo de tratamento proativo, incluindo agendamento da hospitalização materna no mesmo centro e parto cesáreo efetivo. Início imediato de infusão de prostaglandina em baixa dose, e realização da operação de3 a 5 dias mais tarde, coadunam-se com as recomendações de diretriz intrenacional.^2^

O protocolo cirúrgico incluiu a tática de deixar o tórax aberto e o uso de *shunt* de Sano. É interessante notar que o diâmetro do *shunt* foi reduzido por ligadura com fio 5-O absorvível, sempre que fosse preciso coibir o fluxo pulmonar excessivo, determinante de instabilidade hemodinâmica. A diálise peritoneal foi implementada na maioria dos bebês.

O suporte com ECMO foi instituído no pós-operatório em 14 dos 80 pacientes (17,5%). Entre os 73 sobreviventes, 13,7% (10 pacientes) necessitaram de ECMO, em comparação com 57,1% (4 pacientes) dos 7 não sobreviventes.

Oito pacientes adicionais morreram no período entre o primeiro estágio e a operação de Glenn, resultando em taxa de sobrevivência inter-estágios de 81,3%. Este resultado também é comparável aos de grandes centros de referência internacionais, onde coexiste uma política alternativa de alta hospitalar seguida de monitoramento domiciliar.^3^ Considero que a taxa de sobrevivência inter-estágios dos autores fala por si só e deve ser vista como uma diretriz inteligente para equipes que operem pacientes oriundos de áreas distantes.

Embora os autores não tenham comentado a respeito, gostaria de destacar que, se a ECMO não estivesse disponível, a taxa de mortalidade em 30 dias para toda a coorte (80 pacientes), provavelmente, teria caído para 21,25%, ainda assim um resultado muito bom, comparável àqueles dos principais centros internacionais com acesso à ECMO.

Esse resultado hipotético merece consideração. Embora o artigo contenha fortes evidências de que o suporte pós-operatório por ECMO seja recurso salvador de vidas, não se deve subestimar a longa e grande experiência anterior dos autores com a operação de Norwood em primeiro estágio, nem o fato das operações terem sido efetuadas em um dos melhores centros de cardiologia do país.

O leitor deve, pois, perceber que o caminho para o sucesso do grupo de da Silva resultou da associação de habilidade cirúrgica, perseverança, trabalho em equipe, protocolo de tratamento ajustado aos pacientes atendidos, quase todos privados, e acesso à ECMO na Instituição. Que receita!

Cabe mencionar que os resultados com a ECMO, apresentados pelos autores, fornecem alavancagem oportuna para a Sociedade Brasileira de Cirurgia Cardiovascular e outras sociedades correlatas, em seus esforços sustentados na tentativa de incorporar a ECMO ao arsenal do SUS.

O fato é que, na atual era de alocação restrita de recursos, políticas de controle de qualidade e vigilância de resultados cirúrgicos por órgãos reguladores de saúde, o SUS fica atrás de vários outros órgãos congêneres, de outros países da América Latina, no que diz respeito ao provimento de ECMO.

Com a melhoria dos resultados do primeiro estágio da operação de Norwood no Brasil, aqui relatados, anseio pelo momento em que os centros regionais de referência do SUS, que cuidam de bebês com SCEH, passarão a dispor de ECMO, na busca de melhores resultados, em igualdade de condições com o setor privado.

É claro que as dimensões continentais do país, as disparidades regionais e os atuais problemas econômicos, sociais e geopolíticos exigem comedimento. Nesse sentido, as diretrizes para a oferta de ECMO, pelo SUS, poderão se beneficiar de comitê “ad hoc” com a sábia participação das já referidas Sociedades médicas.

Isso posto, e respeitando outras possíveis estratégias terapêuticas existentes, prevejo que instituições conveniadas à cirurgia cardiovascular pediátrica pelo SUS, de vários estados brasileiros, treinadas e melhor equipadas, com ECMO, reenvidarão esforços para obter melhores resultados com a operação de Norwood.
