# Supplementary material for: O Primeiro Estágio da Operação de Norwood no Brasil – Elevamos o Patamar
Source: Arq Bras Cardiol. 2022 Jul 29;119(2):292–3. [Article in Portuguese] doi: 10.36660/abc.20220420 (PMC9363069; doi:10.36660/abc.20220420)
Supplement: Translation

# Translation: The First Stage Norwood Operation, in Brazil – The Bar Was Raised

**Authors:** Walter Villela de Andrade Vicente

**Affiliations:** 1 Ribeirão Preto SP Brazil Universidade de São Paulo Faculdade de Medicina de Ribeirão Preto, Ribeirão Preto, SP – Brazil

**Keywords:** Heart Defects, Congenital, Hypoplastic Left Heart Syndrome/surgery, Extracorporeal Membrane Oxygenation, Norwood Procedures/surgery

Outcomes of the first stage Norwood operation in Brazil have been scarcely reported. Countrywide dismal results, contrasting to those from the developed world, possibly explain this finding.

Contrariwise to this scenario, in this issue of *The Braz J Cardiol*, da Silva´s group presents their single-center experience comprising the largest series of the first-stage Norwood operation with adjuvant postoperative extracorporeal membrane oxygenation (ECMO) support in our country. Their 30-day 91.3% survival, an outstanding surgical result, is on par with those from just a few international elite institutions where ECMO is also available.^1^

The Rubicon was crossed! Hats off to the authors!

Considering that hypoplastic left heart syndrome (HLHS) is the most prevalent and one of the most challenging forms of a single ventricle, the aforementioned report will resonate in the Brazilian pediatric cardiology community and its referral surgical centers, the Brazilian Universal Health System (SUS), the private medical health sector and the general public.

I enjoyed the paper very much. It entails a retrospective evaluation of 80 pts. (private, n=79; public, n=1) operated on from 2016 through 2019. During the period, ECMO assistance was provided to their base hospital, one of the country´s largest cardiovascular surgery referral centers. The report is meticulous and the data are properly analyzed.

It is noteworthy that the authors acknowledge the fact that the patient series benefitted from the surgical group expertise that was accumulated and refined over a long time, encompassing more than 500 cases. In other words, we are looking at the very tip of da Silva´s iceberg-size experience.^1^

A proactive treatment protocol including scheduled, on-site, maternal admission, and cesarean-section delivery was adopted. Immediate low-dose prostaglandin infusion was started and the operation was carried out 3 – 5 days later, as recommended by international guidelines.^2^

The surgical protocol included an open chest policy and a Sano shunt technique. Of note, the shunt was banded with a 5-O absorbable ligature whenever it was deemed necessary to counteract excessive pulmonary flow with concurrent hemodynamic instability. Peritoneal dialysis was implemented in most babies.

ECMO support was instituted postoperatively in 14 patients (17,5%). Among the 73 survivors, 13.7% (10 pts.) required ECMO, as compared to 57.1% (4 pts.) of the 7 non-survivors.

Eight additional pts. died in the interstage period towards the Glenn operation, resulting in an 81.3% interstage survival rate. This result is also comparable to those from major international reference centers, where a competitive home monitoring surveillance policy coexists.^3^ However, one cannot argue that the author´s interstage survival speaks for itself and should be seen as a savvy policy by teams operating on patients coming from remote places.

Although the authors did not comment on this, I wish to highlight that, were ECMO not available, the 30-day mortality rate for the whole cohort (80 pts.) would have probably reached 21.25%, yet a very satisfactory result, comparable to those from international leading centers with handy access to ECMO.

This hypothetical result is worth mentioning though. Although the paper adds strong evidence for postoperative ECMO support as a lifesaving resource, one must not downplay the authors’ previous long and large experience with the first stage Norwood operation, nor the fact that it took place in one of the nation´s best cardiology centers.

The reader should thus perceive da Silva´s group’s pathway to success as resulting from the association of surgical skill, perseverance, teamwork, fitting treatment protocol, and private institutional provision of ECMO runs. Quite a recipe!

Properly considered, the ECMO results presented by the authors provide timely leverage to the Brazilian Society of Cardiovascular Surgery and other related Societies, in their sustained efforts trying to incorporate ECMO into the SUS armamentarium.

The fact is that, in the present era of constrained resource allocation, quality control policies, and surgical results surveillance by health care regulators, the SUS lags behind many other South American countries’ national health administration agencies concerning ECMO backup.

As the bar for the first-stage Norwood operation, in this nation, is now raised, I envision that the SUS regional reference centers caring for HLHS babies be granted ECMO equipment to pursue outcome improvements under equipoise with the private sector.

Of course, the country´s continental size, the regional disparities, and the present economical, geopolitical, and worldwide health distress require pondered consideration. Accordingly, guidelines for SUS ECMO provision should benefit from an “ad hoc” committee with the wise participation of the aforementioned professional Societies.

That happening, and respecting other possible management strategies, I foresee trained and staffed SUS pediatric cardiovascular surgery affiliated institutions, from many Brazilian states, renewing efforts aiming for better outcomes with the Norwood operation, as well.
